# Detection and Quantification of *Leptospira interrogans* in Hamster and Rat Kidney Samples: Immunofluorescent Imprints versus Real-time PCR

**DOI:** 10.1371/journal.pone.0032712

**Published:** 2012-02-29

**Authors:** Adenizar D. Chagas-Junior, Caroline L. R. da Silva, Luciane Marieta Soares, Cleiton S. Santos, Carlos D. C. M. Silva, Daniel A. Athanazio, Mitermayer G. dos Reis, Flávia W. Cruz McBride, Alan J. A. McBride

**Affiliations:** 1 Gonçalo Moniz Institute, Oswaldo Cruz Foundation, Ministry of Health, Salvador, Bahia, Brazil; 2 Health Sciences Institute, Federal University of Bahia, Salvador, Bahia, Brazil; 3 Biotechnology, Centre for Technology Development, Federal University of Pelotas, Pelotas, Rio Grande do Sul, Brazil; Monash University, Australia

## Abstract

A major limitation in the clinical management and experimental research of leptospirosis is the poor performance of the available methods for the direct detection of leptospires. In this study, we compared real-time PCR (qPCR), targeting the *lipL3*2 gene, with the immunofluorescent imprint method (IM) for the detection and quantification of leptospires in kidney samples from the rat and hamster experimental models of leptospirosis. Using a virulent strain of *Leptospira interrogans* serovar Copenhageni, a chronic infection was established in the rat model, which were euthanized 28 days post-infection, while the hamster model simulated an acute infection and the hamsters were euthanized eight days after inoculation. Leptospires in the kidney samples were detected using culture isolation, qPCR and the IM, and quantified using qPCR and the IM. In both the acute and chronic infection models, the correlation between quantification by qPCR and the IM was found to be positive and statistically significant (*P*<0.05). Therefore, this study demonstrates that the IM is a viable alternative for not only the detection but also the quantification of leptospires, particularly when the use of qPCR is not feasible.

## Introduction

Leptospirosis is an emerging neglected disease and is a major threat to public health, especially in developing and under-developed countries [1], [2], [3]. The global burden of leptospirosis has been estimated to be 500,000 cases per year [2], [4], although this is probably under-estimated due to the lack of coordinated surveillance programs and poor diagnosis [5]. The gold standard method for the detection of pathogenic *Leptospira* spp. is culture isolation (CI), however it has poor sensitivity, is hampered by the slow growth of leptospires (requiring four to six months incubation [6]) and there is a high risk of culture contamination [7]. Direct detection by darkfield microscopy is even less sensitive and often results in false-positives due to misinterpretation [8]. The use of PCR, conventional or real-time (qPCR), for the detection of *Leptospira* spp. has resulted in major improvements in specificity and sensitivity [9]. Nevertheless, the widespread application of PCR for the detection of leptospires has been hampered by the risk of contamination with exogenous DNA and the associated risk of false-positives [10], plus reports of variable sensitivity [11].

Previous qPCR assays targeted genes common to all *Leptospira* spp., including *rrs* (16S rDNA) [12], *gyrB* [13], and *secY* [9] genes, or pathogen-specific genes including *lipL32* [14], *ligA* and *ligB* [15]. The *lipL32* gene, which encodes the immunodominant lipoprotein located in the leptospiral outer membrane, is highly conserved among the pathogenic serovars and is absent in the saprophytes [16], [17]. These assays have been used to monitor renal colonization in experimental infection [15], [18], to evaluate urinary shedding of leptospires in dogs [19] and for case confirmation in human subjects during outbreak investigations [20], [21], [22].

In the evaluation of vaccine candidates and leptospiral-host interactions, the detection and quantification of the leptospires is essential. qPCR has become the standard molecular tool for quantification purposes due to its high sensitivity [18]. However, not all laboratories have access to qPCR technology and the standard microbiological methods for quantification are not applicable to the pathogenic *Leptospira* spp. [7]. We previously developed an immunofluorescent imprint method (IM) for the direct detection of pathogenic *Leptospira* spp. by microscopy [23]. This technique is used routinely for detecting the presence of leptospires in the experimental models of leptospirosis used in our laboratories [24], [25], [26]. The aim of this study was to compare the IM with the standard method for quantification of leptospires, qPCR.

## Methods

### Ethics Statement

The Ethical Committee of the Oswaldo Cruz Foundation (Fiocruz) approved the animal protocols used in this study.

### *Leptospira* strain and culture conditions

Leptospires were cultivated in liquid Ellinghausen–McCullough–Johnson–Harris (EMJH) medium (Becton Dickinson and Company, Franklin Lakes, NJ) at 29°C and counted in a Petroff–Hausser counting chamber. A highly virulent isolate from Brazil, *L. interrogans* serogroup Icterohaemorrhagiae serovar Copenhageni strain Cop, was used in all assays. The strain was passaged in hamsters four times and virulent isolates from kidney samples were cultured *in vitro* and stored at −70°C, as previously described [7]. Frozen aliquots were thawed and passaged in EMJH medium up to 14 times prior to use as a virulent isolate in the infection experiments. In previous experiments, the virulence of this strain was evaluated in hamsters and the LD_50_ was calculated to be ∼164 leptospires [24].

### Experimental models of leptospirosis

Laboratory animals (n = 23), the rat and hamster models of leptospirosis, were used in these experiments. Twelve, four-five week-old female Wistar rats (*Rattus norvegicus*, Fiocruz) were infected intraperitoneally with 10^8^ leptospires and were euthanized 28 days post-infection (pi) as described previously [27]. Ten, nine week-old female golden Syrian hamsters (Fiocruz) were infected intraperitoneally with 500 leptospires (3×LD_50_) in 1 ml PBS, and euthanized 8 days pi. A hamster injected with PBS served as the negative control.

### Collection of tissue samples and DNA extraction

Once euthanized, the abdominal cavity was opened and the kidneys were removed aseptically. Good laboratory practice was used in order to avoid DNA cross-contamination (including the use of a laminar airflow bench) and negative controls were included during all the DNA extraction procedures and qPCR steps. Total genomic DNA was extracted from approximately 25 mg tissue, using the QIAamp DNA Mini Kit (Qiagen, São Paulo, SP, Brazil). The tissue sample was a longitudinal section of the kidney that included the cortex and medulla regions, the same section was used in the IM method. The concentration of DNA obtained from tissues was determined with a spectrophotometer (NanoDrop ND 1000, NanoDrop products, Wilmington, DE).

### Culture isolation of leptospires

CI was performed as previously described [27]. Briefly, whole kidney samples were homogenized in 5 ml EMJH, cell debris was allowed to settle for 10 min and 0.5 ml cleared homogenate was used to inoculate 5 ml EMJH. The cultures were incubated at 29°C and were examined regularly for growth, by darkfield microscopy, for up to 8 weeks.

### Imprint detection

Imprints were produced by direct contact of the longitudinally cut surface of the kidney sample, the same region as used in the qPCR assay, onto a glass slide as described previously [23]. Briefly, the kidney imprints were dried, fixed in acetone for 3 min and incubated for 60 min with a primary rabbit polyclonal anti-leptospiral antibody at a dilution of 1∶200. After washing in PBS, the imprints were incubated with a goat anti-rabbit IgG-FITC conjugate at a dilution of 1∶500, washed in PBS and dried before visualization of stained organisms by fluorescence microscopy. Leptospires were quantified in imprint samples as the mean number of leptospires per 10 fields of view at a magnification of 1000×. Only intact spiral-shaped organisms were included in the calculation.

### Real-time quantitative PCR

The *lipL32* gene was amplified using a previously described qPCR assay [19], with the following modifications. The qPCR reaction was performed using an Applied Bioscience 7500 thermocycler and the TaqMan Universal PCR Master Mix (Applied Biosystems, São Paulo, SP, Brazil). The standard curve was prepared from a *L. interrogans* serovar Copenhageni strain Cop culture (2×10^9^ leptospires), centrifuged for 15 min at 10,000× g at 4°C. The recovered pellet was resuspended in PBS and washed by centrifugation (2×15 min, 10,000× g, 4°C). DNA was extracted from the pellet using a QIAamp DNA Mini Kit (Qiagen), as per the manufacturer's instructions. The concentration of the extracted DNA was calculated by spectrometry, optical density 260 and 280 nm (NanoDrop ND 1000), the standard curve was constructed by serial dilutions of the DNA stock. The samples were tested in duplicate, as was each dilution of the standard curve. Each run included a no-template negative control. Results were expressed as the number of genome equivalents per µg kidney DNA [18].

### Statistical analysis

Statistical analyses were performed using the Prism v5 software package (GraphPad Software Inc., La Jolla, CA). The correlation between the methods was compared using the non-parametric Spearman's rank correlation (*r_s_*), *P* values<0.05 were considered significant.

## Results and Discussion

The end-point in the rat model of leptospirosis was a chronic non-lethal infection, as previously reported [27], [28]. As expected, no deaths were observed, the animals were euthanized on day 28 pi and kidney samples were collected for evaluation by CI, IM and qPCR. In contrast, the hamsters developed an acute lethal leptospirosis and in previous reports we observed that symptoms/deaths due to leptospirosis typically occur from day 8 pi onwards [23], [29]. Therefore, the hamsters were euthanized on day 8 pi and kidney samples were collected for evaluation of the presence and quantification of leptospires. All three methods were able to detect leptospires in the kidneys of all of the infected hamsters (10/10) and between 58 (7/12, qPCR) and 67% (8/12, CI and IM) of the infected rats (Table 1). Of note, two of the rats failed to establish a chronic infection. The uninfected controls were negative for the presence of leptospires.

**Table 1:** Comparison of culture isolation (CI), the imprint method (IM) and real-time PCR (qPCR) for the detection of leptospires in animal models simulating chronic (rat) and acute (hamster) infection.

Animal model	Days post-infection	% Leptospire positive (No./total)		
		CI	IM	qPCR
Rat	28	66.6 (8/12)	66.6 (8/12)	58.3 (7/12)
Hamster	8	100 (10/10)	100 (10/10)	100 (10/10)

**Figure 1:**
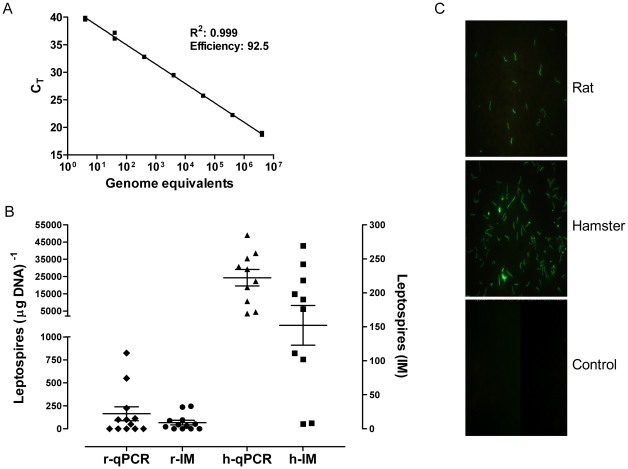
Quantification of leptospires by qPCR and the IM. A. Standard curve of the *lipL32* real-time PCR assay using DNA extracted from ten-fold serial dilutions of an *L. interrogans* strain Cop culture. Each DNA sample was quantified in duplicate and repeated twice. B. Quantification of the leptospiral load in the rat and hamster models. Rats were infected with 10^8^ leptospires and were euthanized on day 28 pi. Hamsters were inoculated with 500 leptospires (3×LD_50_) and euthanized eight days pi. The leptospiral load in the kidneys was determined by qPCR (open symbols) and the IM (solid symbols). The leptospiral loads for the qPCR (leptospires per µg kidney DNA) and the IM (leptospires per 10 fields-of-view, ×1000 magnification) for the rat (r) and hamster (h) are presented as a scatter dot plot of the individual values for each animal, the horizontal line represents the mean value and the error bars the SEM. C. Representative examples of the imprint slides using kidney samples from an infected rat, a hamster and a non-infected control animal (magnification 1000×).

**Figure 2:**
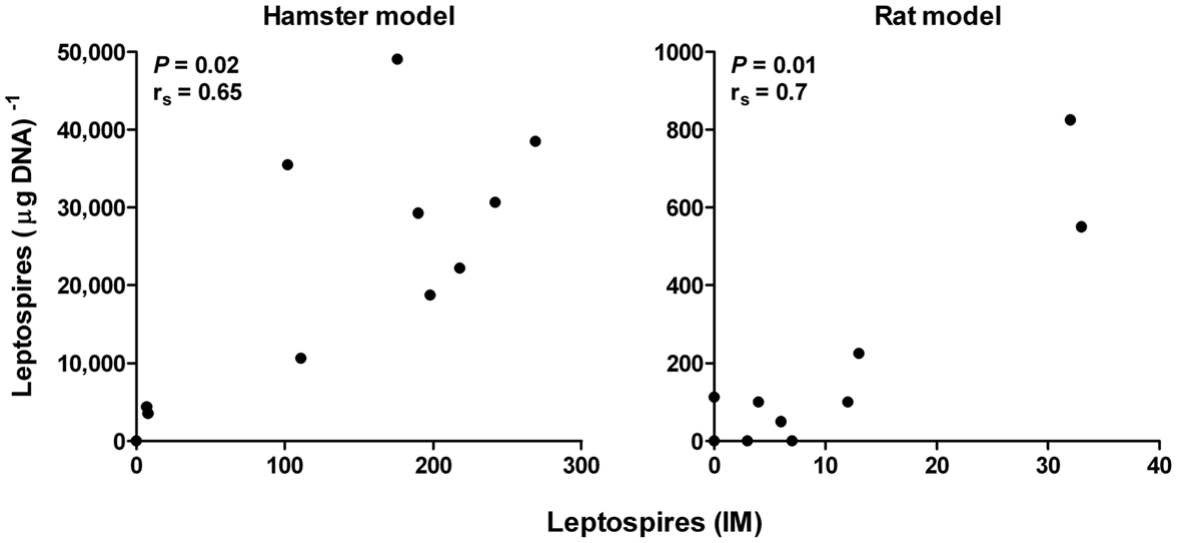
Correlation between the quantification of leptospires by qPCR and the IM. A significant (*P*<0.05), positive correlation was observed between the qPCR and the IM techniques in the experimental models of leptospirosis (rat and hamster) used in this study. The leptospiral loads for the qPCR are displayed as leptospires per µg DNA and in the IM as leptospires per 10 fields-of-view (×1000 magnification).

Quantification of leptospiral load in the animal models was determined by qPCR, based on the assumption of one genome equivalent per spirochaete. The correlation coefficient of the standard curve was 0.999 and the efficiency was 92.4%, Fig. 1A. The limit of detection of the qPCR assay, based on serial dilutions of leptospiral genomic DNA, was estimated to be 4 genome equivalents per reaction or ca. 50 leptospires per µg DNA. This is similar to previous reports for the use of *lipL32* in a qPCR assay [19]. In the hamster model, the leptospiral load ranged from 3.6×10^3^ to 4.9×10^4^ (mean 2.4×10^4^) leptospires per µg DNA and 7 to 269 (mean 138) leptospires in the IM. The qPCR and the IM exhibited a significant positive correlation (*r_s_* = 0.65, *P* = 0.02), see Fig. 2. The leptospiral loads observed among the rats were lower, ranging from 50 to 825 (mean = 163) leptospires per µg DNA and 3 to 33 (mean = 9) leptospires for the qPCR and the IM, respectively. The correlation between the two methods was the highest observed *r_s_* = 0.70, *P* = 0.01, Fig. 2. The correlation coefficients observed in hamsters and rats in this study indicated there was a moderate level of correlation between the methods.

O note, the leptospiral load in the rat model was lower than expected, with a mean of 163 leptospires per µg kidney DNA or a mean 9 leptospires per field-of-view, depending on the method used. Previously, we estimated the leptospiral load in rat kidneys (7–9 days pi) to be ca. 9 leptospires per field-of-view using immunofluorescent microscopy [27], similar to that seen in the current study using the IM. However, as the rat is the one of the main reservoir hosts for urban leptospirosis we expected a higher leptospiral load in the kidneys to allow for excretion to the environment and effective transmission of the disease [30]. A previous report found concentrations of up to 10^7^ leptospires/ml urine 28 days p.i. [31]. A possible explanation is that the higher concentrations of leptospires are found in the renal tubules and not the surrounding kidney tissue in a chronic infection. The methodology used in the current study cannot determine the leptospiral load in renal tubules as the kidney sections used likely included only tubule cross-sections. Indeed, a limitation of the current study is that the concentration of leptospires in the urine of the infected rats was not determined.

The results reported in this study reinforce the usefulness of the IM for the detection of leptospires in commonly used experimental models of leptospirosis and confirm the results of the original imprint study [23]. Since its development, the IM has entered into routine use in our laboratories, in particular for evaluating the carriage status of animals used in the evaluation of potential vaccine candidates. A major drawback of the original study was the lack of a comparison with a qPCR assay to compare sensitivity of detection and quantification of the leptospiral load. This has been addressed in the current study. In terms of detection of leptospires (positive or negative), both the qPCR assay and the IM were comparable to the gold standard method, CI, in the hamster and rat models (Table 1). Note, a potential limitation of the IM and qPCR is their inability to distinguish between viable and non-viable leptospires and this is particularly relevant in determining sterilizing immunity conferred by vaccine candidates.

Another advantage of the IM is the ability to count the leptospires in the imprint samples. However, it was not known how the leptospiral count determined by the IM correlated with the absolute leptospiral load based on qPCR. Therefore, this study evaluated how the two methods covaried by an analysis of correlation in two animal models of leptospirosis. The values determined by qPCR and the IM were analysed for correlation and a significant, positive correlation was observed between the two methods in the hamster and rat models of leptospirosis (Fig. 2). The highest correlation was found in the rat model.

In conclusion, the results of the current study show that for the detection and quantification of leptospires the IM is equivalent to qPCR. In both acute and chronic infection models, the correlation between the IM and the qPCR methods was moderate. The imprint is a detection method that is cheap and is easily established in the laboratory. Furthermore, the fact that only intact leptospires are counted in the IM improves the probability that the observed leptospires are viable. Consequently, the IM is a valuable tool for use in evaluating secondary end-points, such as sterilizing immunity, during vaccine candidate trials and in determining the presence of leptospires in clinical samples.
